# Stem Cell Enrichment with Selectin Receptors: Mimicking the pH Environment of Trauma

**DOI:** 10.3390/s130912516

**Published:** 2013-09-17

**Authors:** Thong M. Cao, Michael J. Mitchell, Jane Liesveld, Michael R. King

**Affiliations:** 1 Department of Biomedical Engineering, Cornell University, Ithaca, NY 14850, USA; E-Mails: tc436@cornell.edu (T.M.C.); mjm529@cornell.edu (M.J.M.); 2 School of Medicine and Dentistry, University of Rochester Medical Center, Rochester, NY 14642, USA; E-Mail: jane_liesveld@urmc.rochester.edu

**Keywords:** CD34+ hematopoietic stem cells, L-selectin, biomimetic, acidic pH

## Abstract

The isolation of hematopoietic stem and progenitor cells (HSPCs) is critical for transplantation therapy and HSPC research, however current isolation techniques can be prohibitively expensive, time-consuming, and produce variable results. Selectin-coated microtubes have shown promise in rapidly isolating HSPCs from human bone marrow, but further purification of HSPCs remains a challenge. Herein, a biomimetic device for HSPC isolation is presented to mimic the acidic vascular microenvironment during trauma, which can enhance the binding frequency between L-selectin and its counter-receptor PSGL-1 and HSPCs. Under acidic pH conditions, L-selectin coated microtubes enhanced CD34+ HSPC adhesion, as evidenced by decreased cell rolling velocity and increased rolling flux. Dynamic light scattering was utilized as a novel sensor to confirm an L-selectin conformational change under acidic conditions, as previously predicted by molecular dynamics. These results suggest that mimicking the acidic conditions of trauma can induce a conformational extension of L-selectin, which can be utilized for flow-based, clinical isolation of HSPCs.

## 1. Introduction

Human hematopoietic stem and progenitor cells (HSPCs) originating from the bone marrow (BM) play a critical role in treating many hematological malignancies due to their unique multipotent, stem cell quality [1,2]. In cancer patients with depleted immune cells, HSPCs transplantation is used to repopulate blood cell lineages [3,4]. On the other hand, increasing evidence has shown that mutagenesis which occurs during the development of HSPCs converts these cells to leukemic stem cells [5,6]. Therefore, a reliable and simple means for the acquisition and enrichment of HSPCs for both transplantation therapy and to better understand leukemia is needed.

HSPCs routinely leave the BM to enter the circulatory system and distant tissues to establish and maintain hematopoiesis [7–10]. During embryogenesis, HSPCs migrate to the fetal liver and differentiate [11–13]. In adults, HSPCs participate in the innate immune response against foreign antigens [14–16]. HSPCs express a repertoire of surface ligands that include unique markers as well as markers shared with leukocytes and circulating tumor cells [17,18] that can bind the family of adhesion molecules called selectins (E-, L- and P-), which facilitates their migration from (and to) the BM and distant tissues [19–23]. In a recent study, we observed that acidic extracellular pH enhances L-selectin:PSGL-1 interactions under flow [24]. Extracellular pH becomes acidic during the early stages of wound healing and inflammation [25,26], which is also a period of elevated recruitment of HSPCs to target sites [27,28]. It follows that HSPCs may experience altered adhesion due to L-selectin:ligand binding in acidic environments.

In this study, we determined that L-selectin ligands expressed on the surface of HSPCs bind with enhanced affinity to L-selectin under acidic extracellular pH. Furthermore, the enhanced L-selectin:ligand binding affinity is due to L-selectin undergoing conformational change in acidic pH as quantified by dynamic light scattering measurements of selectin-PEG-decocorated liposomes. Finally, by mimicking this physiological phenomenon, we demonstrate its potential use to capture and enrich HSPCs by perfusing a suspension of BM cells through L-selectin coated microtubes under acidic pH.

## 2. Experimental Section

### 2.1. Reagents and Antibodies

Phosphate-buffered saline (PBS) and Hank's balanced salt solution (HBSS) were purchased from Invitrogen (Grand Island, NY, USA). Recombinant human P-, L-, and E-selectin/IgG chimera were purchased from R&D Systems (Minneapolis, MN, USA). Phycoerythrin (PE)-conjugated mouse anti-human CD34 (clone 581) and PE-conjugated mouse IgG1 κ-isotype control were purchased from Biolegend (San Diego, CA, USA). APC-conjugated mouse anti-human L-selectin (clone DREG-56) was purchased from BD Biosciences (San Jose, CA, USA).

### 2.2. Isolation of Bone Marrow Cells

Bone narrow mononuclear cells (MNCs) were extracted from consenting adult donors following a protocol approved by the Research Subjects Review Board of the University of Rochester, as described previously [22]. Briefly, bone marrow samples were diluted 3-fold (vol/vol) in Ca^2+^ and Mg^2+^-free PBS, with 35 mL of the diluted sample carefully layered over 15 mL Ficoll cell separation solution (GE Healthcare, Piscataway, NJ, USA) in 50 mL Falcon tubes. Samples were then centrifuged at 800 *g* for 20 min at RT to separate bone marrow MNCs from excess cells and tissue debris. The buffy coat of MNCs was extracted and place into a separate tube and washed twice in PBS. Bone marrow MNCs were quantified and placed in flow buffer (PBS supplemented with Ca^2+^) for flow-based assays.

### 2.3. Isolation of CD34+ Population using Microbeads

To characterize the rolling characteristics of CD34+ HSPCs in acidic pH, CD34+ bone marrow HSPCs were isolated using EasySep Human CD34 Positive Selection Kit by StemCell Technology (Vanvouver, BC, Canada) per manufacturer's instructions. Briefly, a solution of mononuclear cells was incubated with tetrameric antibody complexes against CD34 for 15 min, followed by incubation with dextran-coated magnetic nanoparticles (MNP) for 10 min. The cell-containing tube was then placed in an EasySep® magnet for positive selectin, allowing the MNP-conjugated CD34+ cells to remain in the tube while the supernatant was poured off. The cell population was washed and the magnetic separation was repeated until the desired purity was achieved.

## 2.4. Microtube Functionalization

Micro-renathane (MRE) tubes (300 μm inner diameter, 50 cm long; Braintree Scientific, Braintree, MA, USA) were sterilized with 80% ethanol for 10 min. The tubes were then washed (3×) with PBS buffer (Ca^2+^-free). The inner surface was functionalized with recombinant human L-selectin/Fc at specified concentration for 2 h. The microtubes were then incubated in PBS supplemented with dry milk (5% *w/v*; Sigma-Aldrich, St. Louis, MO, USA) for 1 h to prevent nonspecific adhesion. All steps were performed at room temperature (RT). In several experiments microtubes were labeled with APC conjugated mouse anti human L-selectin for 30 min. Microtubes were washed three times with buffer and images were acquired on an inverted research microscope (Olympus America, Melville, NY, USA).

### 2.5. Flow-Based Cell Adhesion Assay

Cells suspended in PBS buffer (supplemented with 2 mM Ca^2+^) at a specified pH (6.6 or 7.4) were perfused through functionalized microtubes using a syringe pump at a wall shear stress of 2.0 dynes (dyn)/cm^2^. Videos of rolling cells were captured and analyzed using ImageJ (US National Institutes of Health, Bethesda, MD, USA). Cell rolling velocity was determined by measuring the displacement of a rolling cell over time, while rolling flux was determined by quantifying the number of rolling cells entering the image frame over the course of 1 min.

### 2.6. CD34+ HSPC Flow-Based Isolation

A cell suspension of bone marrow MNCs (5 × 10^6^ cell/mL) in PBS buffer (supplemented with 2 mM Ca^2+)^ at specified pH was perfused through L-selectin functionalized microtubes at a wall shear stress of 1.0 dyn/cm^2^. To collect captured cells, the microtube was incubated with fresh PBS buffer (Ca^2+^ free and supplemented with 2 mM of EDTA) for 15 min, and then the cell gently collected into a 1.5 mL Eppendorf tube. Captured cells were also detached from the surface via air embolism. An air bubble is introduced into the microtube using an empty syringe. The bubble is then slowly pushed through the entire length of the microtube to dislodge any remaining captured cells into the collecting tube at the opposing end.

### 2.7. Preparation of Selectin-Conjugated Liposomes

Multilamellar liposomes were prepared using a thin lipid film hydration method as previously described [29,30]. Briefly, lipids were hydrated in 125 mM ammonium sulfate (Sigma-Aldrich) to form multilamellar liposomes, followed by 10 freeze-thaw cycles and then extrusion as previously described [31,32] to prepare unilamellar liposomes. Recombinant human E-, L-, and P-selectin/Fc chimera (rhE/Fc) (R&D Systems, Minneapolis, MN, USA) was conjugated to 1,2-distearoyl-sn-glycero-3-phosphoethanolamine-N-maleimide 2000 (DSPE-PEG2000 maleimide) (Avanti Polar Lipids, Alabaster, AL, USA) via thiolation, and PEG or selectin-PEG conjuguates were covalently attached to diluted unilamellar liposomes as described previously [33]. All liposomes were stored at 4 °C for no more than one week until usage.

### 2.8. Dynamic Light Scattering

To detect changes in selectin protein conformation, freshly prepared selectin-conjugated liposomes (<24 h) were diluted (1,000×) in buffer at specified pH. To remove aggregates, samples were filtered through a 0.45 μm filter (MicroLiter Analytical Supplies, Inc., Suwanee, GA, USA). Samples were analyzed for changes in particle size and polydispersity index (PDI) using a Malvern Zetasizer Nano-ZS (Malvern, Worcestershire, UK).

### 2.9. Flow Cytometry

Isolated cells were stained with mouse anti-human CD34 (clone 581), or mouse IgG1 κ-isotype purchased from Biolegend (San Diego, CA, USA). Cells were washed twice with PBS (supplemented with 1% BSA) and then incubated with antibody at 4 °C for 30 min. Cells were washed twice with buffer and analyzed using a Guava EasyCyte flow cytometer (Millipore, Billerica, MA, USA). CD34+ post-isolation cell populations were quantified and plotted using FlowJo software (Treestar Inc., San Carlos, CA, USA).

### 2.10. Statistical Analysis

Cell rolling velocity and flux were plotted and statistically analyzed using Prism (GraphPad Software, San Diego, CA, USA). Two-tailed unpaired *t*-test was used to determine statistical significance.

## 3. Results and Discussion

### 3.1. CD34+ Human BM Cell Interaction with L-Selectin is Enhanced under Acidic Extracellular pH

**Figure 1.**
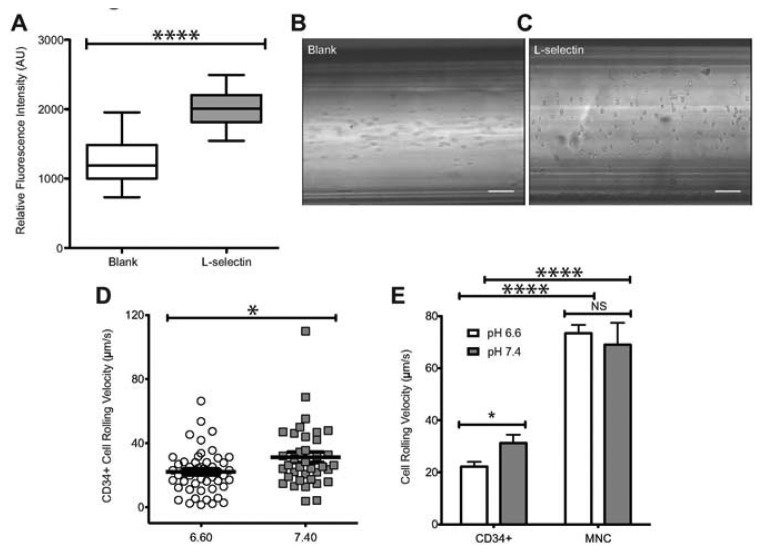
Enhanced adhesion of CD34+ cells to L-selectin at acidic pH. (**A**) Relative fluorescence intensity of L-selectin coated and blank microtubes labeled with APC-anti human L-selectin. (**B**–**C**) Images of perfused cells interacting with blank or functionalized microtubes, respectively. Scale bars are 100 μm. (**D**) Rolling velocity of CD34+ cells under normal (7.4) and acidic (6.6) pH. CD34+ cells at a concentration of 1 × 10^6^ cells/mL were perfused through L-selectin coated (20 μg/mL) microtubes at a shear stress of 2.0 dyn/cm^2^ in buffer at specified pH. (**E**) Comparison of rolling velocities of CD34+ cells and MNCs (unpaired t-test, error bars indicate standard error of the mean; * *p* < 0.05, **** *p* < 0.0001; *n* = 3).

**Figure 2.**
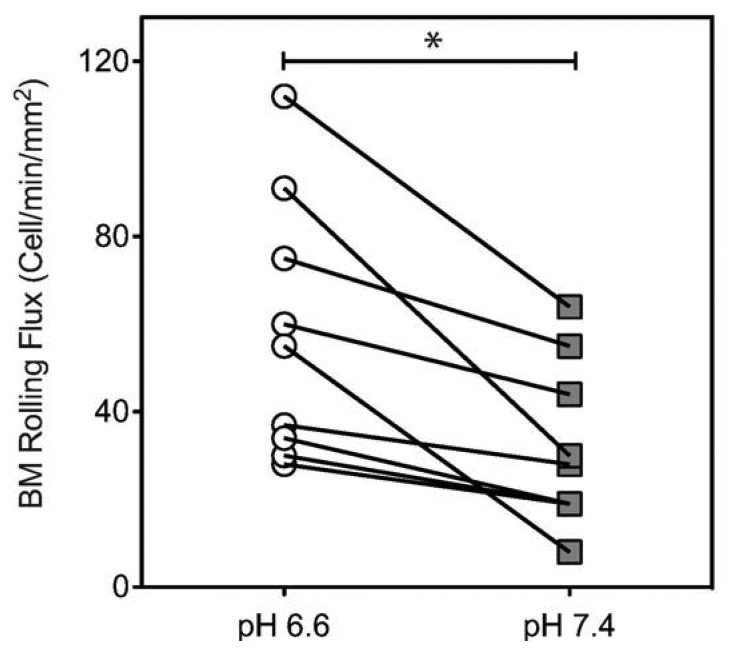
MNCs isolated from bone marrow display higher binding affinity to L-selectin in acidic pH. A suspension of MNCs (1 × 10^6^ cells/mL) was perfused through L-selectin coated (20 μg/mL) microtubes at a shear stress of 2.0 dyn/cm^2^ in buffer at specified pH. Cell rolling flux was measured by counting the number of rolling cells crossing into the image frame over 1 min (unpaired t-test, error bars indicate standard error of the mean; * *p* < 0.05; *n* = 3).

To characterize the influence of acidic extracellular pH on the interaction of human HSPCs and L-selectin, CD34+ cells were perfused through microtubes coated with L-selectin (Figure 1A) at 2 dyn/cm^2^. This level of shear stress was chosen because it is within the physiological shear stress range that mononuclear cells experience in the human circulatory system. CD34+ cells exhibited a significantly lower rolling velocity on L-selectin under acidic conditions (Figure 1D), when compared to L-selectin mediated CD34+ cell rolling at a pH of 7.4. At pH 6.6, CD34+ cells had an average rolling velocity of 22.14 ± 1.87 μm/s, compared to an average rolling velocity of 31.24 ± 3.23 μm/s at pH 7.4. This indicates that under acidic conditions, CD34+ HSPCs experience enhanced binding to L-selectin. To show that the observed interaction between the perfused cells and the coated microtube is L-selectin:ligand specific, cells were perfused through microtubes coated with milk alone. Cell adhesion was not observed in this case (Figure 1B). In contrast, microtubes coated with L-selectin showed extensive cell rolling and adhesion (Figure 1C). In addition, Ca^2+^-dependent cell rolling [34,35] was abrogated by perfusion with HBSS (Ca^2+^ free buffer supplemented with 2mM EDTA) (data not shown) thus confirming that cell interaction was mediated specifically by L-selectin:ligand adhesion. The average rolling velocity of CD34+ cells was significantly lower compared to MNCs from bone marrow (Figure 1E). This observation supports previous work, which suggested that CD34+ cells have stronger binding affinity to L-selectin than CD34- cells [36]. In contrast, no significant differences in CD34- cells were observed under physiological and acidic pH conditions (Figure 1E). An increase in cell rolling flux of MNCs in acidic pH was also found, in comparison to the cell flux measured at physiological pH (Figure 2). These results suggest that acidic pH can be utilized to enhance the number of cell interactions with the L-selectin coating, thus improving the number of cells captured.

### 3.2. Acidic pH Induces Extended Conformation of L-Selectin

**Figure 3.**
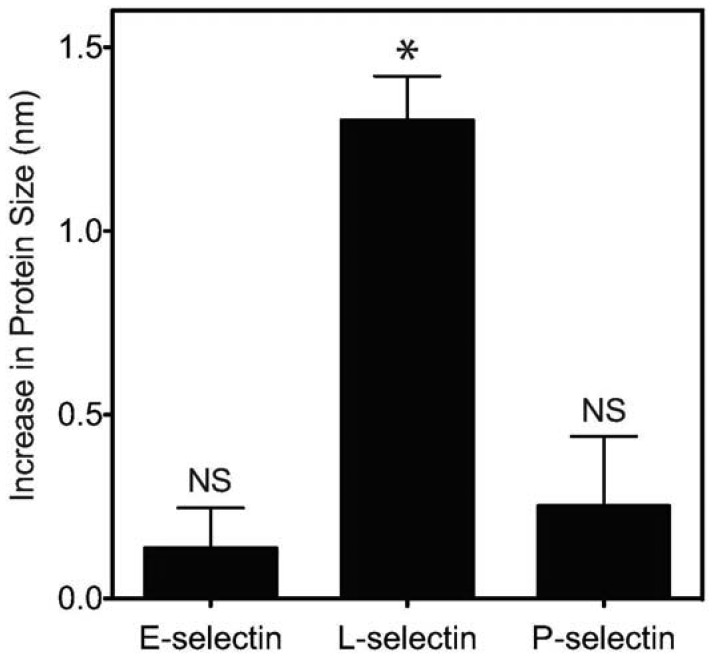
Extension of E-, L-, and P-selectin protein size (in nanometers) upon exposure to acidic (pH 6.6) conditions. Changes in E-, L-, and P-selectin protein size were determined using dynamic light scattering by subtracting the mean particle radius of selectin-coated liposomes under neutral conditions from the mean particle radius of selectin-coated liposomes under acidic conditions. * *p* < 0.05. NS = not significant.

Previous work showed that L-selectin can adopt an “extended” (high affinity) conformation with a point mutation of an amino acid in the EGF domain of L-selectin [37]. This extended conformation results in decreased cell rolling velocity, and an increase in cell flux on the L-selectin ligand PSGL-1. Furthermore, it was previously established that pH can encourage L-selectin to adopt this extended, high affinity conformation due to the abolition of hydrogen bonding between the EGF and lectin domains of L-selectin which normally confines the protein in the “low affinity” conformation [24]. Therefore, we sought to determine whether acidic pH can induce a measurable, extended conformation of L-selectin. Dynamic light scattering (DLS) was utilized to determine changes in the protein size of selectins (E, P and L) presented on nanoscale liposomes in buffer at specified pH. While liposomes in the absence of selectin protein (Table 1) or conjugated with E- or P-selectin exhibited minimal, non-significant changes in hydrodynamic radius (Figure 3), L-selectin significantly increased its average length by 1.3 nm (Figure 3, Table 1) as evidenced by an increase in hydrodynamic radius.

**Figure 4.**
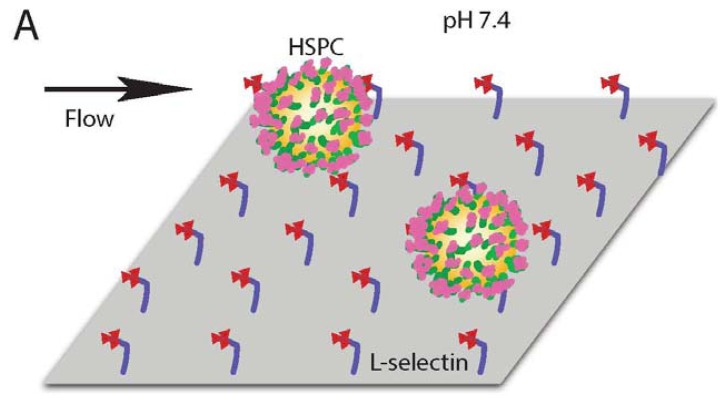

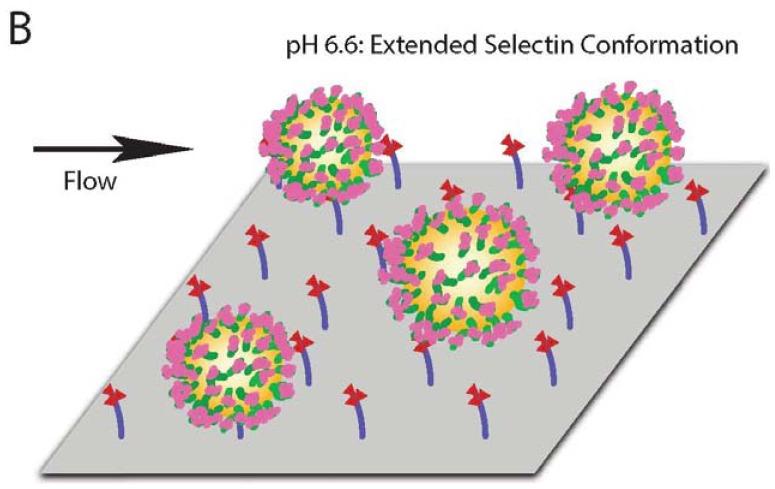
Schematic of increased HSPC adhesion to L-selectin in high affinity, extended conformation under acidic pH (**B**) compared to the lower affinity, bent conformation (**A**).

Together, these results indicate that, in comparison to physiological pH, L-selectin can extend its conformation under acidic pH, which is consistent with an observed enhancement in CD34+ cell adhesion. An extended conformation of L-selectin can allow the protein to bind to its ligands more strongly and at higher frequency, as evidenced by a lower CD34+ cell rolling velocity and increased flux (Figure 4).

### 3.3. L-Selectin Coated-Microtube Captures Human BM CD34+ HSPCs

**Figure 5.**
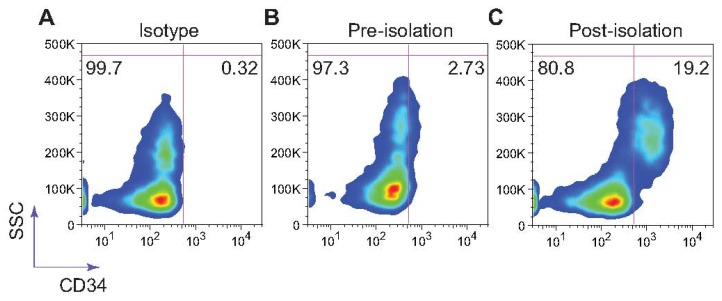
L-selectin mediated isolation of CD34+ cells from patient bone marrow samples under acidic pH. Captured cells were labeled using a mouse anti-human CD34 monoclonal antibody. Flow cytometry plots are a representation of experiments done in triplicate. SSC = side scatter.

Selectin-coated microdevices have been shown to effectively capture viable stem cells [38,39] and circulating tumor cells from whole blood with high yield [40]. To mimic the physiological phenomenon of L-selectin:ligand interaction under acidic conditions for the isolation and enrichment of CD34+ bone marrow cells, low-density bone marrow cells isolated from healthy adult donors using Ficoll were perfused at a concentration 5 × 10^6^ cells/ml through L-selectin coated microtubes (50 μg/mL) at 1.0 dyn/cm^2^ in PBS buffer supplemented with 2 mM Ca^2+^. Adherent cells were dislodged from the surface using both buffer supplemented with 2 mM EDTA and air embolism. Isolated cells were stained using an anti-CD34 monoclonal antibody. L-selectin coated microtubes were found to capture and enrich CD34+ HSPCs from the bone marrow at >19% purity (Figure 5).

## 4. Conclusions

During tissue inflammation, extracellular pH can become increasingly acidic. Furthermore, it is also known that HSPCs are recruited to sites of inflammation via selectin-mediated cell rolling. In this study, we showed that acidic extracellular pH enhances CD34+ HSPCs adhesion to L-selectin, consistent with a measurable extended conformational change of L-selectin to a “high affinity” orientation in acidic pH. This conformational change is taken to increase the frequency of L-selectin:ligand binding. These biophysical insights were applied to the isolation and enrichment of CD34+ HSPCs from bone marrow using an L-selectin coated microtube. The described biomimetic technique allows for both rapid and simple isolation of viable CD34+ HSPCs from patient bone marrow.

## Figures and Tables

**Table 1.** Mean particle radius and polydispersity index (PDI) measurements of selectin-coated liposome samples under neutral and acidic conditions. Data reported as mean ± standard deviation. Results recorded in triplicate.

**Lyposome Type**	**Radius (nm) pH 6.6**	**PDI pH 7.4**	**Radius (nm) pH 7.4**	**PDI pH 6.6**
**PEG only**	52.99 ± 0.95	0.103 ± 0.008	52.33 ± 0.83	0.102 ± 0.013
**PEG + ES**	74.86 ± 1.18	0.101 ± 0.008	74.06 ± 0.94	0.105 ± 0.007
**PEG + LS**	63.08 ± 0.94	0.102 ± 0.011	61.10 ± 0.99	0.101 ± 0.007
**PEG + PS**	84.57 ± 0.83	0.108 ± 0.007	83.65 ± 1.17	0.106 ± 0.009
